# Long-term outcomes of pharmacotherapy in patients with persistent postural-perceptual dizziness

**DOI:** 10.3389/fneur.2025.1566898

**Published:** 2025-03-19

**Authors:** Chihiro Yagi, Akira Kimura, Ryota Kai, Tatsuya Yamagishi, Shinsuke Ohshima, Shuji Izumi, Arata Horii

**Affiliations:** Department of Otolaryngology Head and Neck Surgery, Niigata University Graduate School of Medical and Dental Sciences, Niigata, Japan

**Keywords:** persistent postural-perceptual dizziness, functional disorder, antidepressant, treatment response, long-term outcomes

## Abstract

**Introduction:**

Persistent postural-perceptual dizziness (PPPD) is a chronic neuro-otologic disorder characterized by vestibular symptoms such as dizziness, unsteadiness, or non-spinning vertigo. Its pathophysiology is presumed to involve sensory reweighting to visual/somatosensory sensations for maintaining spatial orientation. Serotonergic antidepressants are a major treatment option for PPPD. However, no reports describe the long-term outcomes of these therapeutic agents in patients with PPPD. Therefore, we evaluated the efficacy of antidepressants administered for up to 3 years after initiation in patients with PPPD.

**Methods:**

Forty-three patients with PPPD (12 men and 31 women; median age at the start of treatment: 49 years) who were started on antidepressants at our department between July 2018 and February 2023 were enrolled. The Dizziness Handicap Inventory (DHI), Hospital Anxiety and Depression Scale (HADS), and Niigata PPPD Questionnaire (NPQ) were used as evaluation measures. Scores at 3 and 6 months and at 1, 1.5, 2, 2.5, and 3 years after starting the medication were compared with pre-treatment scores. Furthermore, head-tilt perception gain (HTPG), an indicator of somatosensory hypersensitivity, was measured before and after treatment.

**Results:**

Significant improvements in all DHI, HADS, and NPQ measures were observed from 3 months onward and were maintained at all timepoints up to 3 years after treatment. Meanwhile, there was no significant improvement in HTPG after medication treatment was initiated. Patients whose DHI scores improved by ≥18 points were considered treatment responders; 27 of the 43 patients were responders at 1 year after treatment. During the initial 2 weeks of treatment, adverse events, including nausea/abdominal distension, were observed in 26 patients; however, the adverse events did not last until the subsequent observation timepoint. During the case enrollment period, antidepressants were started in 59 patients, and 43 patients included in the present study were able to continue medication (overall adherence rate: 72.9%).

**Conclusions:**

The long-term efficacy and safety of serotonergic antidepressants were demonstrated in patients with PPPD. However, somatosensory hypersensitivity, which is sometimes observed as a clinical feature in patients with PPPD, did not improve after pharmacotherapy. Developing treatments to reduce hypersensitivity may improve treatment outcomes.

## 1 Introduction

Persistent postural-perceptual dizziness (PPPD) is a chronic neuro-otologic disorder characterized by vestibular symptoms such as dizziness, unsteadiness, or non-spinning vertigo (1). The pathophysiology of PPPD is believed to involve sensory reweighting in response to visual/somatosensory sensations to maintain spatial orientation (2, 3), and hypersensitivity to sensory input is one of the clinical features sometimes observed in patients with PPPD (4, 5). Thus, PPPD is classified as a functional rather than an organic/psychiatric disorder. Although the presence of psychiatric conditions is not necessary for diagnosing PPPD, neuroticism and anxiety are well-known comorbidities (6, 7). Regarding therapy, serotonergic antidepressants are a major treatment option for PPPD (8). To date, there have been two reports demonstrating the efficacy of serotonergic antidepressants for PPPD (9, 10). However, the effectiveness of the antidepressants was evaluated for only up to 3 months after the initiation of treatment. There are no reports on the long-term outcomes of serotonergic antidepressant treatment in patients with PPPD. Therefore, we conducted this study to evaluate the efficacy of antidepressants administered for up to 3 years after initiation in patients with PPPD. In addition, we performed the head roll-tilt subjective visual vertical (HT-SVV) test, which can assess somatosensory hypersensitivity in PPPD (11), before and after the antidepressant treatment to examine whether antidepressants can improve somatosensory hypersensitivity in patients with PPPD.

## 2 Methods

### 2.1 Patients

Patients with PPPD who were started on antidepressants at the Department of Otolaryngology, Head and Neck Surgery at Niigata University Medical and Dental Hospital between July 2018 and February 2023 and had been on the medication for at least 6 months were enrolled in this study. PPPD was diagnosed using the Barany Society criteria (1). Patients were treated only with serotonergic antidepressants, without the inclusion of non-pharmacological therapies such as vestibular rehabilitation or cognitive-behavioral therapy. In terms of antidepressant selection, selective serotonin reuptake inhibitors were chosen in the early phase of the study, serotonin and noradrenaline reuptake inhibitors as well as noradrenergic and specific serotonergic antidepressants in the middle phase, and multimodal antidepressants in the later phase. Consequently, the choice of antidepressants was not differentiated by patient. The exclusion criteria were as follows: patients who had already started antidepressants at other hospitals, non-compliance with the use of antidepressants, and discontinuation of antidepressants due to side effects. The precipitating conditions for PPPD in the study cohort are shown in Supplementary Table 1.

### 2.2 Clinical symptom scales

#### 2.2.1 Dizziness Handicap Inventory

The Dizziness Handicap Inventory (DHI) is a standard 25-question questionnaire designed to quantitatively evaluate the degree of handicap felt by patients with vestibular disorders in their daily lives (12, 13). The total score of the DHI ranges from 0 to 100, with 0 indicating no disability and 100 indicating severe disability.

#### 2.2.2 Hospital Anxiety and Depression Scale

The Hospital Anxiety and Depression Scale (HADS) is a questionnaire that consists of self-administered anxiety and depression subscales. Each HADS subscale includes seven questions (14), and each question is scored on a scale of 0 (not at all) to 3 (most of the time, very often). The total score for each HADS subscale is 21, and the total score for the entire HADS is 42, with higher scores indicating higher levels of anxiety and depression.

#### 2.2.3 The Niigata PPPD Questionnaire

The Niigata PPPD Questionnaire (NPQ) is a self-administered questionnaire used for screening and assessing the severity of PPPD (15). The NPQ consists of 12 questions that assess the degree of exacerbation of symptoms for three exacerbating factors: upright posture or walking, active or passive movement, and visual stimulation. The severity of each factor was evaluated using four questions, with the scores for each question ranging from 0 (none) to 6 (unbearable). The total score for each factor is 24, and the total score for the NPQ is 72, with higher scores indicating greater severity.

### 2.3 Head roll-tilt subjective visual vertical test

The Head roll-tilt subjective visual vertical (HT-SVV) (16) test is used for estimating somatosensory hypersensitivity and was performed using the HT-SVV examination system (UNIMEC, Aichi, Japan). In this system, SVV during head roll-tilt can be measured in addition to conventional head-upright SVV (UP-SVV). During the examination, each participant was instructed to sit on a chair in front of a bar-display box and wear goggles to exclude any visual information other than that in the bar-display box. The head roll-tilt angle (HTA), which is the tilt angle of the head relative to the gravity axis, was measured using a linear accelerometer attached to the goggles. The participants were instructed to adjust the oblique bars on the display back to the vertical position using a keypad, either in the head-upright (=0° tilt) or head roll-tilt (−30° or 30° tilts) condition. The SVV was measured 14 times (six times for the 0° tilt and four times each for the −30° and 30° tilts) in a pseudo-random order.

The UP-SVV was measured six times in relation to the Earth's vertical, and the average value of the six measurements was used for analysis.

Head-tilt perception (HTP) is defined as the angle between the head tilt and the SVV (Supplementary Figure 1), and can be calculated using the following equation:


(1) HTP(°)= HTA−SVV

The HTPG is the slope in the regression equations for UP-SVV and HT-SVV, with the *x*-axis representing the HTA and the *y*-axis representing the HTP. An HTPG value > 1.0 indicates that the participant felt and recorded a greater head-tilt angle than the actual; that is, overestimation. An HTPG value < 1.0 indicates the opposite effect; that is, underestimation. The HTPG was calculated for the right and left head tilts to obtain the right and left HTPGs. The average of the right and left HTPGs was defined as the mean HTPG.

### 2.4 Vestibular tests

#### 2.4.1 Bithermal caloric testing

Bithermal caloric testing was performed by stimulating each external auditory canal twice with air at 26°C and 45°C for 60 s at 5-min intervals. The maximum slow phase velocity was measured using electronystagmography, and the presence of canal paresis (CP) (%) was determined using Jongkee's index formula (17). A CP value of 20% or higher was considered indicative of significant unilateral caloric weakness.

#### 2.4.2 Cervical- and ocular vestibular-evoked myogenic potentials

Cervical- and ocular vestibular-evoked myogenic potentials (cVEMP and oVEMP) were used to assess saccular and utricular function, respectively, and were measured using the Neuropack^®^ system (Nihon Koden, Tokyo, Japan). The click (0.1-ms rarefactive square waves of 105-dB nHL) was used to induce cVEMP. A hand-held electromechanical vibrator (Minishaker^®^ Bruel & Kjaer, Nærum, Denmark) fitted with a short bolt terminating in a plastic cap was used to record oVEMP. The vibrator delivers a 500-Hz tone burst (4-ms plateau and 1-ms rise and fall) on a patient's skull at the Fz (midline of the hairline). Amplitudes and latencies were measured at the response peaks, which occurred at approximately 13 and 23 ms for cVEMP and 10 and 15 ms for oVEMP, depending on the stimulus. The difference between the peak amplitudes was used to determine the peak-to-peak (PP) amplitude. To compare the two ears, the asymmetry ratio (AR) was calculated using the following formula: (right – left)/(right + left) x 100 (%) on the raw PP amplitude (18). The upper limit of the AR was set at 34.0 for cVEMPs (19) and 34.4 for oVEMPs (20).

### 2.5 Statistical analysis

DHI, HADS, and NPQ scores recorded at 3 and 6 months and at 1, 1.5, 2, 2.5, and 3 years after starting the medication were compared with the pre-treatment scores. The observation period for this study was set until October 2024. Regarding the HT-SVV test, the pre-treatment UP-SVV and mean HTPG were compared with the values recorded ~1-year post-treatment. The Wilcoxon signed-rank sum test was used for the analysis of UP-SVV, mean HTPG, and DHI, HADS, and NPQ scores.

Patients were defined as responders if their DHI scores decreased by 18 or more points at 6 months to 1 year after starting the medication. The designation of a patient as a responder was based on the minimum detectable change in DHI, i.e., the minimum change that was considered significant, which is 18 points (21). Comparisons of sex, age, disease duration, and each pre-treatment score (DHI/HADS/NPQ) between responders and non-responders were performed. Chi-square test was used for the analysis of sex-specific differences, and the Mann–Whitney U test was used to analyze age, disease duration, and DHI, HADS, and NPQ scores. Multiple logistic regression analysis was performed to identify the predictive factors for treatment response.

All statistical analyses were performed using Graph Pad Prism version 9 (GraphPad Software, San Diego, CA, USA). Statistical significance was set at *p* < 0.05.

### 2.6 Ethics statement

This study was approved by the institutional review board of Niigata University Medical and Dental Hospital (Niigata City, Japan) (#2024-0092). The board waived the requirement for informed consent owing to the retrospective design of the study. Patients were provided information about the study and offered the opportunity to refuse participation. All the study procedures were performed in accordance with the ethical standards of the 1964 Helsinki Declaration.

## 3 Results

### 3.1 Demographic and clinical characteristics

Forty-three patients with PPPD (12 men and 31 women) were included in the present study. The median age of the patients was 49 years [interquartile range (IQR): 26 years] (Table 1). The median duration from disease onset to the start of medication was 16 months (IQR: 43 months). Regarding comorbidities, four patients had hyperlipidemia, two each had hypertension and endometriosis, and one each had diabetes, functional hearing loss, myasthenia gravis, somatic symptom disorder, and migraine unrelated to vestibular symptoms. The median total DHI, HADS, and NPQ scores before treatment were 61.0 (IQR: 32.0), 20.5 (IQR: 10.5), and 42.5 (IQR: 22.8), respectively. The median CP value was 12.3% (IQR: 22.5%). The median asymmetry ratios of cVEMP and oVEMP were 16.0% (IQR: 20.4%) and 11.1% (IQR: 17.4%), respectively. Abnormal responses were observed in the caloric testing of 10 patients (24.4%), the cVEMP of 7 patients (18.4%), and the oVEMP of 5 patients (16.7%). The medications administered included venlafaxine (*n* = 22 patients), escitalopram (*n* = 10 patients), vortioxetine (*n* = 5 patients), mirtazapine (*n* = 3 patients), and paroxetine (*n* = 3 patients). During the initial 2 weeks of treatment, adverse events, including nausea/abdominal distension, were observed in 26 patients (60.5%); however, the adverse events did not last until the subsequent observation timepoint. Between July 2018 and February 2023, 59 patients were started on antidepressants in our department. Out of these, 43 patients who were included in the present study could continue the medication, resulting in an overall adherence rate of 72.9%.

**Table 1:** Clinical and demographic characteristics of 43 patients with PPPD before treatment.

**Variables**	
Sex, male/female	12/31
Age	49 (26)
Disease duration, month	16 (43)
DHI (total score)	61.0 (32.0)
HADS (total score)	20.5 (10.5)
NPQ (total score)	42.5 (22.8)
CP, % (*n* = 41)	12.3 (22.5)
cVEMP (asymmetry ratio), % (*n* = 38)	16.0 (20.4)
oVEMP (asymmetry ratio), % (*n* = 30)	11.1 (17.4)

Values are reported as medians and interquartile ranges (IQR), apart from the sex ratio, which is reported as an absolute value.

PPPD, persistent postural-perceptual dizziness; HADS, Hospital Anxiety and Depression Scale; DHI, Dizziness Handicap Inventory; NPQ, Niigata PPPD Questionnaire; CP, canal paresis; cVEMP and oVEMP, Cervical- and ocular vestibular-evoked myogenic potentials.

### 3.2 Changes in DHI, HADS, and NPQ scores after treatment

**Figure 1:**
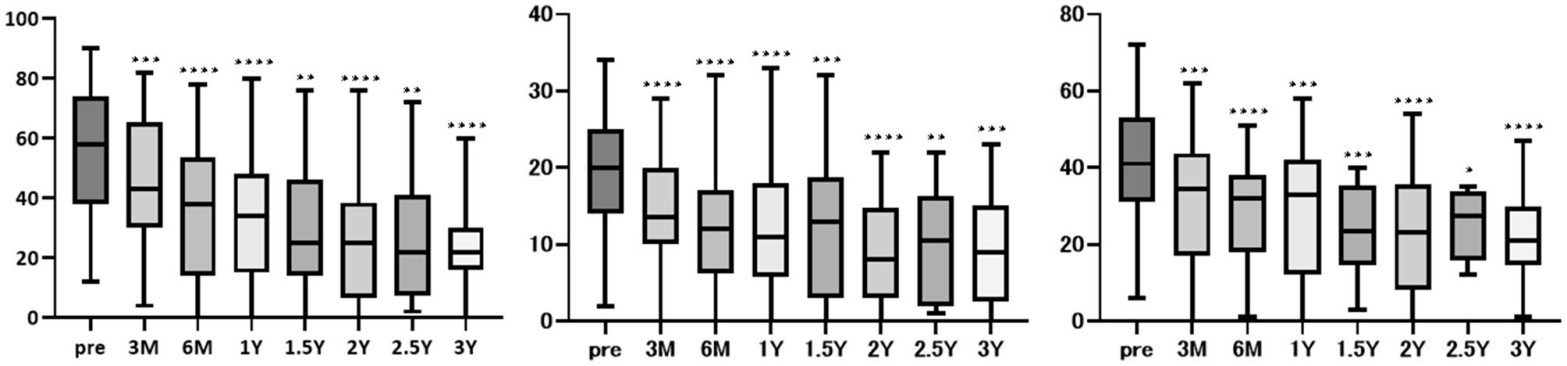
Changes in DHI, HADS, and NPQ scores after treatment. DHI, HADS, and NPQ scores before treatment and up to 3 years post-treatment. Significant improvements in all three measures were observed from 3 months onward and were maintained at all timepoints up to 3 years after treatment.

Comparisons of the pre-treatment DHI, HADS, and NPQ scores with those recorded at 3 and 6 months and 1, 1.5, 2, 2.5, and 3 years after starting the medication are shown in Table 2 and Figure 1. Significant improvements in all DHI, HADS, and NPQ measures were observed from 3 months post-treatment onward and were maintained at all timepoints up to 3 years after treatment. Of the 43 patients, six were referred to other clinics after their symptoms had stabilized; however, their data after referral could not be obtained. Of the 43 patients, 22 were still on medication and were followed up as outpatients up to October 2024. Two patients discontinued the medication because their symptoms improved; however, they resumed the medication due to exacerbation of their symptoms. Ten patients (23.3%) completely discontinued the medication due to improvement of symptoms. Three patients dropped out, and two were placed on other treatments due to their insufficient response to the serotonergic antidepressants. Therefore, the data presented in Table 2 and Figure 1 include not only those of the well-controlled “star” patients but also the data of all followed outpatients. Of the 22 patients who were followed up until October 2024, eight were on the medication for < 3 years, 3 for 3–5 years, and 11 for more than 5 years.

**Table 2:** Changes in DHI, HADS, and NPQ scores after treatment.

**Evaluation timepoint**	***N***	**DHI**	**HADS**	**NPQ**
Pre-treatment	43	61.0 (32.0)	20.5 (10.5)	42.5 (22.8)
**Post-treatment**				
3 M	40	43.0 (35.5)^***^	13.5 (10.0)^****^	34.5 (26.8)^***^
6 M	40	38.0 (39.5)^****^	12.0 (10.8)^****^	32.0 (20.0)^****^
1 Y	38	34.0 (33.0)^****^	11.0 (12.3)^****^	33.0 (29.8)^***^
1.5 Y	20	25.0 (32.0)^**^	13.0 (15.8)^***^	23.5 (17.8)^***^
2 Y	28	25.0 (32.0)^****^	8.0 (11.8)^****^	23.0 (27.5)^****^
2.5 Y	12	22.0 (33.5)^**^	10.5 (14.3)^**^	27.5 (18.0)^*^
3 Y	20	22.0 (14.0)^****^	9.0 (12.5)^***^	21.0 (15.3)^****^

Values are reported as medians and interquartile ranges (IQR). Scores recorded at 3 and 6 months and at 1, 1.5, 2, 2.5, and 3 years after starting the medication were compared with the pre-treatment scores using the Wilcoxon signed-rank sum test.

HADS, Hospital Anxiety and Depression Scale; DHI, Dizziness Handicap Inventory; NPQ, Niigata PPPD Questionnaire.

^*^Values indicate statistical significance.

^*^p < 0.05; ^**^p < 0.01; ^***^p < 0.001; ^****^p < 0.0001.

**Figure 2:**
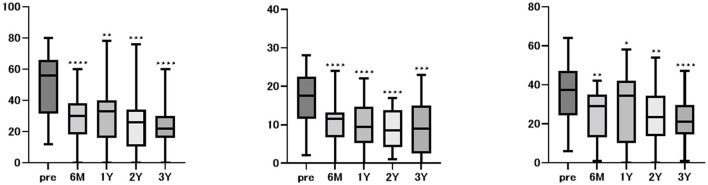
Changes in DHI, HADS, and NPQ scores after treatment in 20 patients over a 3-year follow-up period. DHI, HADS, and NPQ scores were assessed before treatment and up to 3 years post-treatment in 20 patients with a 3-year follow-up. Significant improvements in all DHI, HADS, and NPQ measures were observed at 6 months, as well as at 1, 2, and 3 years after treatment.

Twenty patients who were followed for up to 3 years after starting the medication were selected for subanalysis. Significant improvements in all DHI, HADS, and NPQ measures were observed at 6 months and at 1, 2, and 3 years after treatment (Figure 2).

### 3.3 Comparison of HT-SVV test results before and after treatment

Table 3 shows the comparison of HT-SVV test results before and after treatment in 21 patients with PPPD. DHI, HADS, and NPQ scores improved significantly before and after treatment; however, there were no significant changes in UP-SVV or mean HTPG after treatment.

**Table 3:** Comparison of the HT-SVV test results of 21 patients with PPPD before and after treatment.

**Variables**	**Pre-treatment**	**Post-treatment**	***p*-value**
UP-SVV, degree	1.83 (2.17)	1.17 (1.50)	0.29
Mean HTPG	1.21 (0.16)	1.16 (0.16)	0.32
DHI (total score)	54.0 (33.0)	40.0 (30.0)	< 0.0001^****^
HADS (total score)	17.0 (14.5)	8.0 (7.0)	< 0.0001^****^
NPQ (total score)	36.0 (25.0)	31.0 (31.5)	< 0.01^**^

Values are reported as medians and interquartile ranges (IQR). The Wilcoxon signed-rank sum test was performed to analyze the pre- and post-treatment values.

PPPD, persistent postural-perceptual dizziness; UP-SVV, head-upright subjective visual vertical; HTPG, Head-tilt perception gain; DHI, Dizziness Handicap Inventory; HADS, Hospital Anxiety and Depression Scale; NPQ, Niigata PPPD Questionnaire. ^**^p < 0.01; ^****^p < 0.0001.

### 3.4 Predictors of treatment response

After 1 year of treatment or at 6 months of treatment for patients with missing data on 1-year assessments, 27 of 43 patients (62.8%) were identified as responders, with DHI scores at least 18 points lower than their pre-treatment scores. Table 4 presents the results of the comparison of demographic and clinical characteristics between the responder and non-responder groups. The responder group had significantly higher pre-treatment DHI scores than the non-responder group, with no significant differences in sex, age, disease duration, or pre-treatment HADS and NPQ scores between the two groups. Table 5 displays the results of the multiple logistic regression analysis performed to identify factors that could predict treatment response. Significant differences in pre-treatment DHI scores were observed; however, no other background factors significantly predicted treatment response.

**Table 4:** Demographic and clinical characteristics of responders and non-responders.

**Variables**	**Responders (*n* = 25)**	**Non-responders (*n* = 18)**	***p*-value**
Sex, male/female	7/18	5/13	0.99
Age	47.0 (20.0)	57.5 (29.0)	0.51
Disease duration, month	17.0 (33.0)	14.0 (43.0)	0.92
DHI (total score)	68.0 (28.0)	50.0 (29.0)	< 0.01^**^
HADS (total score)	21.0 (8.0)	17.5 (9.8)	0.25
NPQ (total score)	47.0 (28.5)	38.5 (16.0)	0.38

Values are reported as medians and interquartile ranges (IQR), apart from the sex ratio, which is reported as an absolute value. Fisher's exact test was performed for the analysis of sex, whereas the Mann-Whitney *U*-test was used for the analysis of age, disease duration, and DHI, HADS, and NPQ scores.

DHI, Dizziness Handicap Inventory; HADS, Hospital Anxiety and Depression Scale; NPQ, Niigata PPPD Questionnaire. ^**^p < 0.01.

**Table 5:** Multiple logistic regression analysis of the variables associated with 1-year treatment response in patients with PPPD.

**Predictors**	**OR**	**95% CI**	***p*-value**
Sex, male/female	0.55	0.07–3.66	0.54
Age	0.95	0.89–1.01	0.13
Disease duration, month	1.00	0.98–1.03	0.89
DHI (total score)	1.10	1.04–1.18	< 0.01^**^
HADS (total score)	0.93	0.80–1.06	0.30
NPQ (total score)	0.97	0.91–1.03	0.36

PPPD, persistent postural-perceptual dizziness; OR, odds ratio; CI, confidence interval; DHI, Dizziness Handicap Inventory; HADS, Hospital Anxiety and Depression Scale; NPQ, Niigata PPPD Questionnaire. ^**^p < 0.01.

Of the 16 non-responders, two switched to cognitive-behavioral therapy, and three dropped out. Eleven patients continued to receive the initial antidepressant medication because they experienced some symptom relief, even though their scores did not improve by more than 18 points on the DHI.

## 4 Discussion

### 4.1 Background characteristics and long-term outcomes of pharmacotherapy for patients with PPPD

The clinical and demographic characteristics of the patients with PPPD in the present study are consistent with those described in previous reports (22, 23). Most of the patients were middle-aged women, had the disorder for more than a year, had a high severity of vestibular symptoms (DHI), and had moderately high mental symptom scores (HADS) (Table 1). The percentage of patients with abnormal caloric test results was 24.4%, which is similar to the values reported in previous studies (24, 25). However, the percentage of patients with abnormal VEMP was 16–18%, which is slightly lower than previously reported values (25). Oka et al. (25) reported that the vestibular function of patients with PPPD is affected by preceding disease. In the present study, the number of precipitating conditions that could lead to abnormal vestibular function test results (e.g., vestibular neuritis) was small (Supplementary Table 1), leading to slightly better VEMP results. In summary, the patients in this study had almost typical background characteristics of patients with PPPD.

In the present study, significant improvements in all DHI, HADS, and NPQ measures were observed from 3 months onward and were maintained at all timepoints up to 3 years after treatment (Table 2; Figure 1). Even when treatment efficacy was assessed solely in patients who were followed for 3 years after starting the medication, the effect of serotonergic antidepressants was sustained for up to 3 years later. Although a previous study demonstrated the efficacy of serotonergic antidepressants at 20 weeks and 12 months in patients with chronic subjective dizziness, which is a precursor to PPPD (26), the present study is the first to show the long-term efficacy of antidepressants in patients diagnosed with PPPD based on the diagnostic criteria of the Barany Society. Notably, while the long-term efficacy of the antidepressants was demonstrated in the present study, only 23.3% of the patients exhibited complete improvement of their symptoms and discontinued the medication. This indicates that patients with PPPD may not easily achieve complete remission. Additionally, 11 patients in the present study had been taking the medication for more than 5 years, and all of them were able to continue treatment without adverse events. This suggests that serotonergic antidepressant treatment is safe for the long-term management of PPPD.

### 4.2 Effects of antidepressants on somatosensory hypersensitivity in patients with PPPD

The patients with PPPD showed no significant difference in HTPG on the HT-SVV test before and after antidepressant treatment; however, the treatment effectively resulted in changes in all subjective symptom scales (Table 3). It has been reported that patients with PPPD tend to exhibit hypersensitivity to sensory stimuli, leading to exaggerated responses to sensory input (5). In our previous study, we found that the mean HTPG of patients with PPPD is significantly higher than that of patients with other chronic vestibular disorders (11), a finding considered indicative of somatosensory hypersensitivity. This study demonstrated no significant change in mean HTPG before and after treatment, suggesting that antidepressants do not improve somatosensory hypersensitivity in PPPD. In the present study, the response rate up to 1 year post-treatment was 62.8%, which is comparable to response rates reported in previous studies (50–70%) (10, 27). However, a response rate of about 60% is insufficient, and higher treatment responses could be achieved if therapies aimed at improving somatosensory hypersensitivity are developed in the future.

### 4.3 Predictors of treatment response

In this study, patients with higher pretreatment DHI were more likely to respond to treatment than those with lower scores (Table 5), suggesting that the more severe the dizziness, the more effective the medication was. Meanwhile, no other factors that could predict treatment response were identified in this study. It has been reported that paroxetine is particularly effective for reducing vestibular symptoms in patients with chronic dizziness who have high Zung Self-Rating Depression Scale scores, indicating that more severe mental distress could predict favorable outcomes when treating psychogenic dizziness with antidepressants (28). Conversely, in the treatment of PPPD, even patients with low depression and anxiety scores can expect improvement in dizziness. Therefore, proactive administration of antidepressants should be considered for patients with severe vestibular symptoms and high DHI scores, regardless of comorbid psychiatric conditions. Regarding sex differences, previous reports have indicated that women are more responsive to medication (10), which is inconsistent with the current results; thus, further studies are needed to assess the sex-specific efficacy of pharmacotherapy for PPPD.

### 4.4 Limitations

This study has several limitations. First, it was a retrospective study; therefore, the potential risk of selection bias in the distribution of patients with PPPD cannot be entirely ruled out. Second, the number of patients included in this study was relatively small. Consequently, the number of factors considered in the multiple logistic regression analysis was limited, and the relationships between treatment response and other factors, such as vestibular function or types of antidepressants, were not examined. Third, since no treatment or placebo group was assigned, the possibility of improvement in the absence of treatment or the placebo effect cannot be dismissed.

## 5 Conclusions

This study demonstrated the long-term efficacy and safety of serotonergic antidepressants in patients with PPPD. However, these antidepressants had no effect on somatosensory hypersensitivity, which is sometimes observed as a clinical feature of PPPD. Developing treatments to reduce hypersensitivity may improve treatment outcomes.

## Data Availability

The raw data supporting the conclusions of this article will be made available by the authors, without undue reservation.

## Supplementary material

Supplementary Figure 1 The head roll-tilt subjective visual vertical (16). The HTP is defined as the angle between the perceived head tilt and the SVV. g, gravity; HTA, head roll-tilt angle; HTP, head-tilt perception; SVV, subjective visual vertical.

Supplementary Table 1 Precipitation conditions for persistent postural-perceptual dizziness in 43 patients.

## References

[1] Staab JP Eckhardt-Henn A Horii A Jacob R Strupp M Brandt T. Diagnostic criteria for persistent postural-perceptual dizziness (PPPD): consensus document of the committee for the classification of vestibular disorders of the Barany Society. J Vestib Res. (2017) 27:191–208. 10.3233/VES-170622 29036855 PMC9249299

[2] Qin C Zhang R Yan Z. Research progress on the potential pathogenesis of persistent postural-perceptual dizziness. Brain Behav. (2025) 15:e70229. 10.1002/brb3.70229 39740787 PMC11688117

[3] Madrigal J Herrón-Arango AF Bedoya MJ Chen JC Castillo-Bustamante M. Persistent challenges: a comprehensive review of persistent postural-perceptual dizziness, controversies, and clinical complexities. Cureus. (2024) 16:e60911. 10.7759/cureus.60911 38910644 PMC11193666

[4] Powell G Derry-Sumner H Shelton K Rushton S Hedge C Rajenderkumar D. Visually-induced dizziness is associated with sensitivity and avoidance across all senses. J Neurol. (2020) 267:2260–71. 10.1007/s00415-020-09817-0 32306170 PMC7359147

[5] Hashimoto K Takeuchi T Ueno T Suka S Hiiragi M Yamada M. Effect of central sensitization on dizziness-related symptoms of persistent postural-perceptual dizziness. Biopsychosoc Med. (2022) 16:7. 10.1186/s13030-022-00235-4 35255948 PMC8900397

[6] Yan Z Cui L Yu T Liang H Wang Y Chen C. Analysis of the characteristics of persistent postural-perceptual dizziness: a clinical-based study in China. Int J Audiol. (2017) 56:33–7. 10.1080/14992027.2016.1211763 27686369

[7] Trinidade A Harman P Stone J Staab JP Goebel JA. Assessment of potential risk factors for the development of persistent postural-perceptual dizziness: a case-control pilot study. Front Neurol. (2021) 11:601883. 10.3389/fneur.2020.601883 33551961 PMC7859446

[8] Yagi C Kimura A Horii A. Persistent postural-perceptual dizziness: a functional neuro-otologic disorder. Auris Nasus Larynx. (2024) 51:588–98. 10.1016/j.anl.2023.12.008 38552422

[9] Yu Y-C Xue H Zhang Y-X Zhou J. Cognitive behavior therapy as augmentation for sertraline in treating patients with persistent postural-perceptual dizziness. BioMed Res Int. (2018) 2018:8518631. 10.1155/2018/8518631 29707579 PMC5863356

[10] Min S Kim JS Park HY. Predictors of treatment response to pharmacotherapy in patients with persistent postural-perceptual dizziness. J Neurol. (2021) 268:2523–32. 10.1007/s00415-021-10427-7 33544219

[11] Yagi C Morita Y Kitazawa M Nonomura Y Yamagishi T Ohshima S. Head roll-tilt subjective visual vertical test in the diagnosis of persistent postural-perceptual dizziness. Otol Neurotol. (2021) 42:e1618–24. 10.1097/MAO.0000000000003340 34538854 PMC8584214

[12] Jacobson GP Newman CW. The development of the Dizziness Handicap Inventory. Arch Otolaryngol Head Neck Surg. (1990) 116:424–7. 10.1001/archotol.1990.01870040046011 2317323

[13] Goto F Tsutsumi T Ogawa K. The Japanese version of the Dizziness Handicap Inventory as an index of treatment success: exploratory factor analysis. Acta Otolaryngol. (2011) 131:817–25. 10.3109/00016489.2011.565423 21762004

[14] Zigmond AS Snaith RP. The Hospital Anxiety and Depression Scale. Acta Psychiatr Scand. (1983) 67:361–70. 10.1111/j.1600-0447.1983.tb09716.x 6880820

[15] Yagi C Morita Y Kitazawa M Nonomura Y Yamagishi T Ohshima S. validated questionnaire to assess the severity of persistent postural-perceptual dizziness (PPPD): The Niigata PPPD Questionnaire (NPQ). Otol Neurotol. (2019) 40:e747–52. 10.1097/MAO.0000000000002325 31219964 PMC6641087

[16] Wada Y Yamanaka T Kitahara T Kurata J. Effect of head roll-tilt on the subjective visual vertical in healthy participants: towards better clinical measurement of gravity perception. Laryngoscope Investig Otolaryngol. (2020) 5:941–9. 10.1002/lio2.461 33134543 PMC7585259

[17] Jongkees LB Maas JP Philipszoon AJ. Clinical nystagmography. A detailed study of electro-nystagmography in 341 patients with vertigo. Pract Otorhinolaryngol. (1962) 24:65–93. 10.1159/000274383 14452374

[18] Rosengren SM Colebatch JG Young AS Govender S Welgampola MS. Vestibular evoked myogenic potentials in practice: methods, pitfalls and clinical applications. Clin Neurophysiol Pract. (2019) 4:47–68. 10.1016/j.cnp.2019.01.005 30949613 PMC6430081

[19] Murofushi T Matsuzaki M Wu CH. Short tone burst-evoked myogenic potentials on the sternocleidomastoid muscle: are these potentials also of vestibular origin? Arch Otolaryngol. (1999) 125:660–4. 10.1001/archotol.125.6.660 10367923

[20] Chihara Y Iwasaki S Ushio M Murofushi T. Vestibular-evoked extraocular potentials by air-conducted sound: another clinical test for vestibular function. Clin Neurophysiol. (2007) 118:2745–51. 10.1016/j.clinph.2007.08.005 17905655

[21] Rizk HG Velozo C Shah S Hum M Sharon JD Mcrackan TR. Item level psychometrics of the Dizziness Handicap Inventory in vestibular migraine and Meniere's disease. Ear Hear. (2024) 45:106–14. 10.1097/AUD.0000000000001405 37415269

[22] Azzi JL Khoury M Séguin J Rourke R Hogan D Tse D. Characteristics of persistent postural perceptual dizziness patients in a multidisciplinary dizziness clinic. J Vestib Res. (2022) 32:285–93. 10.3233/VES-190749 34151875

[23] Park JH Nguyen TT Kim SH Park JY Na S Jeon EJ. Clinical characteristics of persistent postural-perceptual dizziness and its visual subtype in Korean patients: a multicenter cross-sectional study. Brain Behav. (2024) 14:e3389. 10.1002/brb3.3389 38391108 PMC10831130

[24] Adamec I Juren Meaški S Krbot Skoric M JaŽic K Crnošija L Milivojevic I. Persistent postural-perceptual dizziness: clinical and neurophysiological study. J Clin Neurosci. (2020) 72:26–30. 10.1016/j.jocn.2020.01.043 31948878

[25] Oka M Ichijo K Koda K Kamogashira T Kinoshita M Igarashi K. Preceding balance disorders affect vestibular function in persistent postural-perceptual dizziness. J Clin Med. (2023) 12:2589. 10.3390/jcm12072589 37048672 PMC10095344

[26] Staab JP Ruckenstein MJ Solomon D Shepard NT. Serotonin reuptake inhibitors for dizziness with psychiatric symptoms. Arch Otolaryngol Head Neck Surg. (2002) 128:554–60. 10.1001/archotol.128.5.554 12003587

[27] Popkirov S Stone J Holle-Lee D. Treatment of persistent postural-perceptual dizziness (PPPD) and related disorders. Curr Treat Options Neurol. (2018) 20:50. 10.1007/s11940-018-0535-0 30315375

[28] Horii A Mitani K Kitahara T Uno A Takeda N Kubo T. Paroxetine, a selective serotonin reuptake inhibitor, reduces depressive symptoms and subjective handicaps in patients with dizziness. Otol Neurotol. (2004) 25:536–43. 10.1097/00129492-200407000-00022 15241233

