# Supplementary figures and images for: Long-term outcomes of pharmacotherapy in patients with persistent postural-perceptual dizziness
Source: Front Neurol. 2025 Mar 19;16:1566898. doi: 10.3389/fneur.2025.1566898 (PMC11961416; doi:10.3389/fneur.2025.1566898)

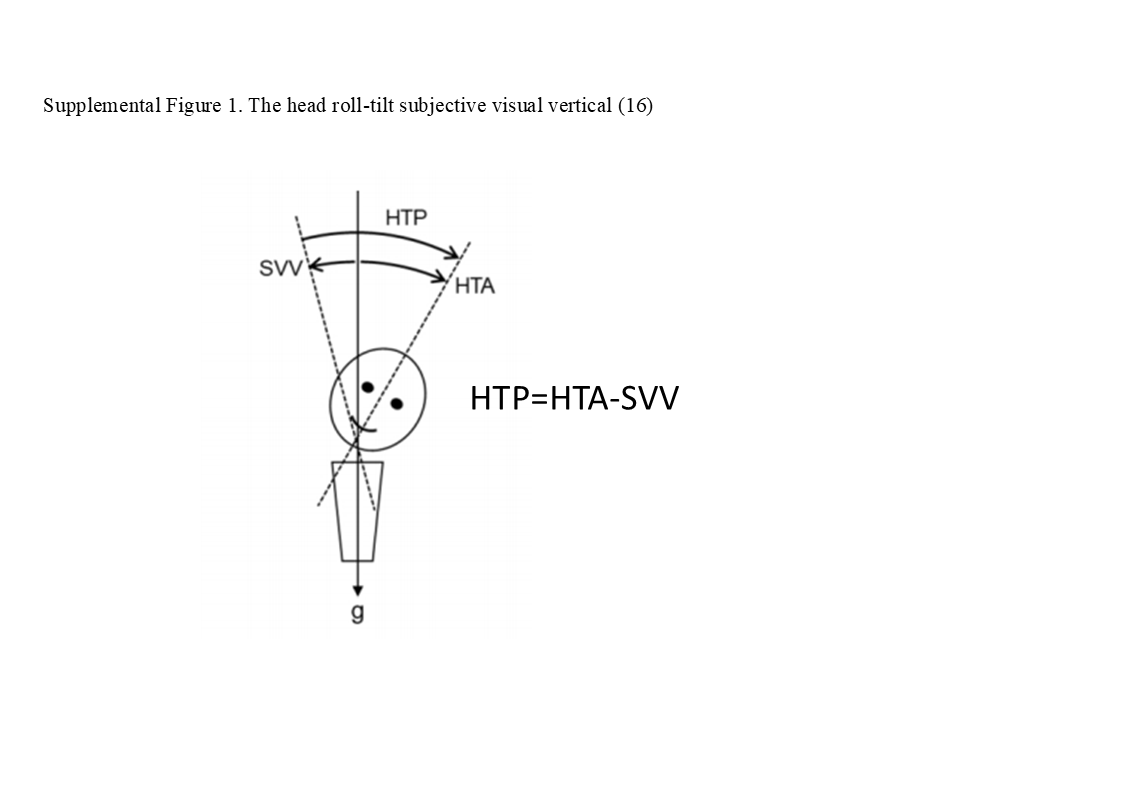

Supplement: Supplementary Figure 1 — The head roll-tilt subjective visual vertical (16). The HTP is defined as the angle between the perceived head tilt and the SVV. g, gravity; HTA, head roll-tilt angle; HTP, head-tilt perception; SVV, subjective visual vertical. [file Image_1.tif]
