# Supplementary material for: Long-term outcomes of pharmacotherapy in patients with persistent postural-perceptual dizziness
Source: Front Neurol. 2025 Mar 19;16:1566898. doi: 10.3389/fneur.2025.1566898 (PMC11961416; doi:10.3389/fneur.2025.1566898)
Supplement: Supplementary Table 1 — Precipitation conditions for persistent postural-perceptual dizziness in 43 patients. [file Table_1.docx]

Supplementary Material

**Supplemental Table 1.** **Precipitation conditions for persistent postural-perceptual dizziness in 43 patients**

| Acute attack of peripheral vestibular vertigo | n = 13 |
| --- | --- |
| BPPV | n = 10 |
| Chronic anxiety disorders | n = 6 |
| Meniere’s disease | n = 4 |
| No specific precipitants | n = 3 |
| Idiopathic sudden sensorineural hearing loss with vertigo | n = 3 |
| Inner ear dysfunction after ear surgery | n = 1 |
| Orthostatic dysregulation | n = 1 |
| Vestibular migraine | n = 1 |
| Vestibular neuritis | n = 1 |

Abbreviations: BPPV, benign paroxysmal positional vertigo
